# Factors associated with quality of life in patients with benign prostatic hyperplasia, 2009–2016

**DOI:** 10.1097/MD.0000000000030091

**Published:** 2022-09-09

**Authors:** Sewon Park, Kyu-Sung Lee, Mankyu Choi, Munjae Lee

**Affiliations:** a Department of Medical Humanities and Social Medicine, Ajou University School of Medicine, Suwon, South Korea; b Department of Medical Device Management and Research, SAIHST, Sungkyunkwan University, Seoul, South Korea; c Department of Urology, Samsung Medical Center, Sungkyunkwan University School of Medicine, Seoul, South Korea; d Department of Health Policy & Management, College of Health Science, Korea University, Seoul, South Korea; e BK21 FOUR R&E Center for Learning Health Systems, Korea University, Seoul, South Korea; f Medical Research Collaborating Center, Ajou Research Institute for Innovative Medicine, Ajou University Medical Center, Suwon, South Korea.

**Keywords:** benign prostatic hyperplasia, health related quality of life, Korea, physical activity, sitting time

## Abstract

This study analyzed the factors affecting the health-related quality of life of patients with benign prostatic hyperplasia (BPH) according to age. We also aimed to determine appropriate strategies to improve their quality of life. Data from the Korea Health Panel Survey (2009–2016) were used in this study. A total of 3806 patients with BPH were divided into 2 groups: younger adults (aged under 65 years) and older adults (aged over 65 years). In addition, a logistic regression analysis was conducted to identify factors affecting the quality of life of young and older patients with BPH. In younger adult patients with BPH, the higher the level of education, the higher the quality of life by a factor of 1.379, and the more intense the physical activity, the lower the quality of life by a factor of 0.791. Also, the longer the sitting time, the lower the quality of life by a factor of 0.765. In contrast, for older adult patients with BPH, the quality of life improved by factors of 1.601 and 2.921, respectively, for health insurance and higher income level. In addition, it was found that the quality of life decreased by a factor of 0.754 in patients who drink alcohol. In order to improve the quality of life of the middle-aged adult population with BPH, it is necessary to reduce sitting time through constant physical activity. Moreover, the cost of medical care should be reduced and the quality of life increased through reductions in surgical treatment, as the burden of medical expenses will degrade the quality of life for older adults.

## 1. Introduction

Benign prostatic hyperplasia (BPH) commonly occurs in men aged > 50 years, and the prevalence increases with age. In patients with BPH, the medical expenditure increases with increasing age and treatment duration.^[1–3]^ Accordingly, to reduce the social costs, which is required for the welfare of the aged society, systematic preventive management and strategic approach to BPH are currently needed.

With BPH, the patient may experience residual urine, frequent urination, intermittent urination, abdominal pressure during urination, nocturnal enuresis, limited movements, sleep disturbance, etc. This provokes mental health problems and causes inconvenience in daily life, resulting in deterioration of the health-related quality of life.^[4]^ Health-related quality of life represents an individual’s subjective assessment and satisfaction on the overall situations, living, and experience of life. In other words, it is a concept that includes the elements needed to understand the satisfaction in an individual’s life. Therefore, the health status of the individual may affect his or her daily life, which also influences his or her satisfaction of life.^[5]^ In addition, as activities of daily living are related to the quality of life and physical activities may improve the mental health and quality of life, restrictions on activities of daily living in patients with BPH seem to affect their quality of life.^[6]^

Although BPH is a disease most frequently affecting older adults, the prevalence of BPH in middle-aged men over 30 has continuously increased, and the number of patients visiting hospitals due to the inconvenience in daily life is also increasing.^[7]^ In this study, we aimed to analyze the factors influencing the health-related quality of life of patients with prostatic hypertrophy according to age and then to understand the quality of life of patients with BPH. Furthermore, we seek measures to prevent BPH and improve patients’ quality of life.

## 2. Methods

### 2.1. Data source

This study used the integrated data from the Korea Health Panel over the span of 8 years from 2009 to 2016. The Korea Health Panel Survey (KHPS) is the Government-Approved Statistical Survey (No. 92012) where the Korea Institute for Health and Social Affairs and the National Health Insurance Corporation organize a consortium to yield baseline data, which includes the use of people for health and medical care services, expenditure levels, health behaviors, etc. This data is a cohort DB that has been implemented every year since 2008 and is being investigated based on a sample extracted by the probability proportional stratification method using the 2005 Population and Housing Survey as the population. As of 2008, it consisted of 7866 households and 21,283 people nationwide, and 2222 households were added as a new sample in 2013 to compensate for the sample dropout problem. For the extracted sample households, a household survey that can confirm household characteristics such as household income and housing type, and a household member survey that investigates demographic characteristics such as age, education level, marital status, and medical use histories such as hospitalization, outpatient treatment, and emergency is composed of Since 2009, subjective health status and health behavior-related contents have been additionally investigated, so data from 2009 were used in this study.

**Figure 1.**
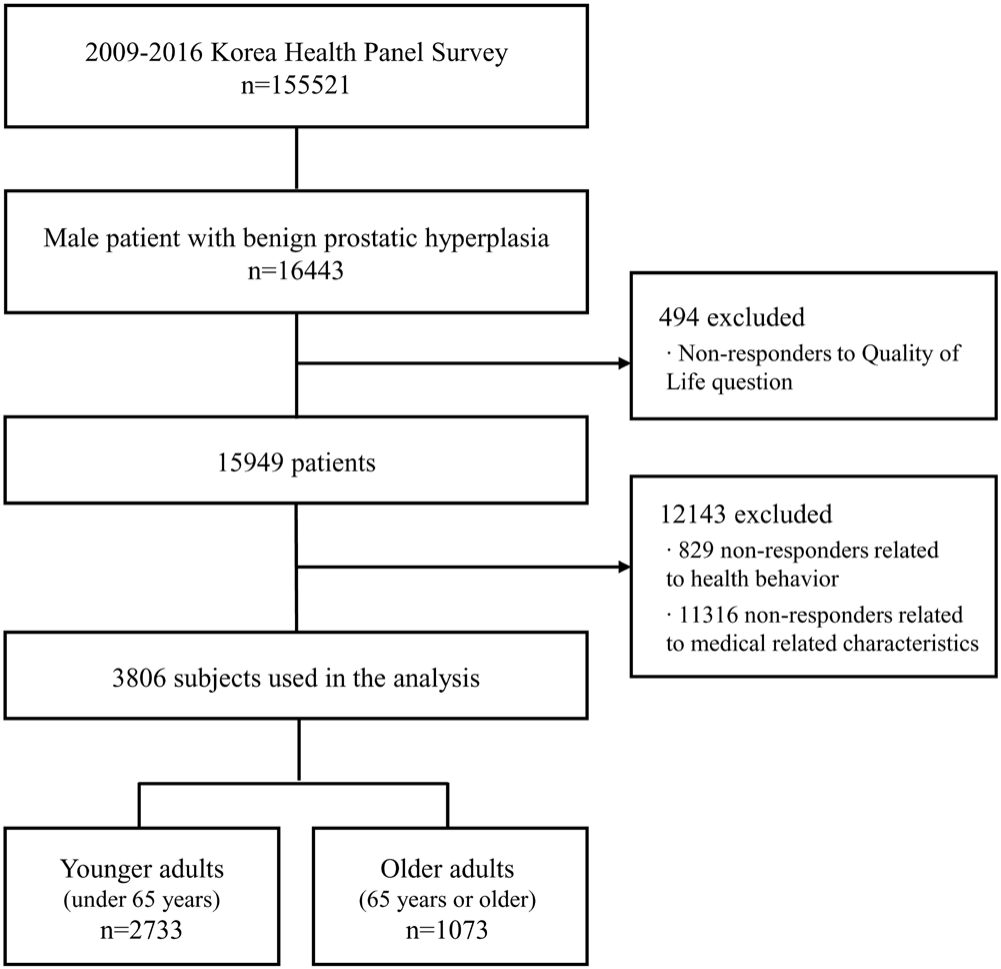
Flowchart.

Of the variables used in the analysis, quality of life was measured consistently during the measurement period in 2009–2013 and in 2015–2016, except for 2014. The items measuring time spent sitting were gauged from 2011 to 2016. In addition, the items that measured unmet medical care were not gauged in 2010, and consistently measured for the intervening years. In certain years, some variables were not measured. It was judged that some bias could occur if the analysis would be conducted excluded the data of an entire year. Therefore, in this study, data such as the annual demographic characteristics, medical use status, and health behavior of household members from 2009 to 2016 were merged based on the household member identification number to construct panel data for 8 years. According to the data type, panel data can be divided into balanced panels and unbalanced panels. A balanced panel is data in which all cross-sectional data are observed over the entire period, and data that is not in a balanced panel is called an unbalanced panel. In this study, an imbalanced panel was constructed to include as many study subjects as possible and to specifically identify factors affecting the quality of life (Fig. 1).

First, since BPH is known to be a common disease in men, 16,443 males who were diagnosed with BPH were extracted as the research subjects. The definition of study subjects is as follows. KHPS comprehensively grasps the status of disease and morbidity, medical conditions, and health behaviors of households and household members. In particular, the types of medical use are classified into emergency, hospitalized, and outpatient. Because most patients with chronic diseases have high outpatient use, this study analyzed by using outpatient data. For analysis, patients with BPH outpatient were extracted. The diseases of the genitourinary system investigated by the KHPS include acute nephritic syndrome, renal failure, urinary stone, and urethral stenosis. Among them, patients with outpatient visits due to BPH were extracted. Next, patients who answered that the doctor had diagnosed with BPH were extracted. Even if the reason for the medical institution’s visit is BPH, the doctor’s diagnosis may appear differently. Finally, patients who were diagnosed by a doctor and regularly treated for BPH were extracted. In order to understand the prevalence of patients diagnosed with BPH, the patient who answered yes to the question “Are you visiting the hospital to manage and treat BPH?” was finally defined as a patient with BPH.

Second, except the subjects (n = 494) who did not respond to the measurement questions on quality of life, those (n = 829) who did not answer the questions on health behavior, and those (n = 11316) who did not respond to the questions on medical care-related properties, a total of 3806 people were selected as the research subjects. Among patients diagnosed with BPH and treated regularly, patients who responded to questions related to the quality of life and health behavior were extracted were used for analysis. In particular, in the case of measuring the quality of life, we tried to more specifically utilize factors affecting the quality of life of patients with BPH by using the items measured by patients undergoing treatment to manage BPH. Lastly, in order to analyze the 3806 patients with BPH, they were divided into 2 groups: younger adults and older adults. People under 65 years were classified as younger adults (n = 2733) and people over 65 years as older adults (n = 1073).

### 2.2. Description of variables

#### 2.2.1. Participants’ general characteristics.

The variables used in this study were as follows: age, education level, income and forms of medical security. Generally, people over 65 are referred to as the elderly, and according to the World Health Organization (WHO), people over 65 are defined as the elderly.^[8,9]^ In terms of education level, the participants were grouped into those who graduated high school and those who did not graduate high school. In terms of income, the participants were divided into groups based on the annual gross household income: those who earned more than 2,400USD/month and those who earned <2,400USD/month. Korea’s median income is 2,400USD/month, so it is classified based on 2,400USD/month.^[10]^ Since the medical costs related to the treatment of BPH tends to increase over time, analyzing the quality of life according to the type of medical security is necessary. Medical security refers to the systematic provision of the necessary health and medical services required to protect the people’s right to health and includes National health insurance, medical benefits, and Industrial Accident Compensation Insurance. In this study, the form of health insurance was divided into health insurance, which is a public assistance system, and medical benefits for low-income people. Therefore, medical security types were classified into health insurance subscribers and medical beneficiaries.

#### 2.2.2. Health behavior and medical-related characteristics of participants.

Subjective health status, unmet medical care needs, chronic disease, physical activity, time spent sitting, smoking, and drinking were used as variables associated with the health behaviors and medical-related characteristics of participants. Regarding subjective health status, the questionnaire items in the KHPS were utilized, which were rated on a 5-point scale. 1 point means very good, 2 point means good, 3 point means average, 4 point means bad, and 5 point mean very bad. In other words, the higher the score, the higher the negative effects on health. Therefore, 1–3 points were subjective health status as “good”, and 4–5 points were subjective health status as “bad”. Unmet medical care needs were divided into presence and absence of unmet medical care needs, based on the participants’ response to the following question: “In the previous year, did you have any experience that you have needed to receive treatments or examinations in clinics and hospitals but you could not?” In addition, even those who had at least 1 chronic disease were classified as patients with chronic disease.^[11,12]^ By calculating the amount of energy used during physical activities into metabolic equivalent task (MET)-minutes according to the energy requirements of the respondents, which were determined based on their response to the survey, physical activities were classified into intense and moderate. Sedentary lifestyle is known to be a bad habit that lowers blood circulation and stimulates the prostate due to compression of the perineum and is a risk factor for BPH due to reduced physical activity.^[13]^ Because the risk of BPH increases in those who sit for a long time, we included time spent sitting as a variable. National Health Statistics indicate that the daily sitting time of the remaining hours, excluding the average 7 hours of sleep, is 8 hours for men and 7.8 hours for women; since this study was only conducted on men, daily sitting time was given an 8-h threshold.^[14,15]^ For smoking, we used smoking experience and current smoking status. Patients who smoked in the past but not currently or have never smoked were classified as nonsmokers, and those who smoked every day or sometimes were classified as smokers. In the case of patients who smoked in the past but not currently were defined, it was judged that there was no problem in classifying them as nonsmokers because only those who had quit smoking for 3 years or more were included. Regarding drinking status, patients who have not drunk in the past 1 year were classified as nondrinkers, while the remaining were classified as drinkers. In this study, since the purpose of this study was to identify the factors affecting the quality of life according to whether or not drinking of BPH, it was classified into drinkers and nondrinkers by using drinking status in the questionnaire.^[16,17]^

#### 2.2.3. Health-related quality of life.

Health-related quality of life was analyzed using the score measured by the European Quality of life-5 Dimension (EQ-5D) scores in the KHPS. EQ-5D has been included in the KHPS since 2009 and is being investigated. The health-related quality of life items that the KHPS is investigating include motor ability, self-management, daily activities, pain/inconvenience, and anxiety/depression. Each question is composed of a 3-point scale: “not troubled,” “somewhat troubled,” and “troubled.” ^[18]^ EQ-5D can be expressed as a single score between 0 and 1 by assigning a weight, and the closer it is to 1, the better the quality of life. Based on the health-related quality of life, the measurement items were treated as 0 points (troubled) and 1 point (not troubled) in order to classify them into deteriorated group and non- deteriorated groups of quality of life.^[19–22]^

### 2.3. Statistical analysis

In this study, we conducted an analysis using the SPSS 25.0 Program. First, frequency analysis was performed to identify the sociodemographic characteristics of patients with BPH in younger adults and older adults. Second, independent sample t-tests were implemented to analyze the differences in the quality of life according to the participants’ general characteristics, health behaviors, and medical-related characteristics according to age. Third, logistic-regression analysis was executed to determine the factors affecting the quality of life of patients with BPH in younger adults and older adults. To conduct this analysis, sociodemographic characteristics, medical use-related characteristics, and health behaviors were set as independent variables; the deteriorated group (0) and nondeteriorated group (1) in the health-related quality of life were used as dependent variables. Published Korea Health Panel Survey (KHPS) was used for the analysis, and ethical approval was not required for this study. No prior informed consent was required from the participants as the participant information was completely anonymized and not identified prior to analysis.

## 3. Results

### 3.1. Characteristics of participants

The research subjects in this study were 3806 patients with BPH: 2733 patients (71.8%) under 65 years and 1073 patients (28.2%) over 65 years. The general characteristics of the subjects are shown in 1. In terms of educational attainment, 92.8% of patients in the younger adult group graduated from at least the high school level, whereas in the older adult patients, 56.1% did not graduate from high school, suggesting a low education level. Based on income level, the number of people in the bracket for who earn less than $ 2400 was the highest for both young & middle-aged adults (1452 people, 53.1%) and older adults (807 people, 75.2%). Based on their type of medical security, both young & middle-aged adults and older adults were health insurance subscribers - 2701 people (98.8%) and 1047 people (97.6%). Based on subjective health status, the patients who thought that their health status was not good accounted for 12.9% in younger adults and 33.9% in older adults; the patients who experienced unmet medical care was 27.8% in younger adults and 25.3% in older adults. The number of people without chronic diseases was determined to be 2005 (73.4%) and 789 people (73.5%) for younger adults and older adults, respectively; suggesting that most patients classified as older adults had chronic diseases. Among those that did not participate in moderate physical activities, there were 1791 people (65.5%) in the younger adults and 722 people (67.3%) in the older adults. However, the number of people not involved in intense physical activities was 2136 people (78.2%) in younger adults and 922 people (85.9%) in older adults; through which it could be seen that most patients with BPH were less physically active. Most of both younger adults and older adults had a sitting time of less than 8 hours, at 71.3% and 77.3%, respectively. In addition, both younger adults and older adults were nonsmokers (1400 people [51.2%] and 764 people [71.2%], respectively). Based on drinking habits, 89.3% of younger adults and 66.8% of older adults drank alcohol (Table 1).

**Table 1:** Characteristics of participants (n = 3806).

Characteristic	Categories	Younger adults (under 65 years)		Older adults (65 years or older)	
		N	%	N	%
Age		2733	71.8	1073	28.2
Education	<High school	198	7.2	602	56.1
	≥High school	2535	92.8	471	43.9
Income	<$ 2400	1452	53.1	807	75.2
	≥$ 2400	1281	46.9	266	24.8
Type of medical security	National health insurance	2701	98.8	1047	97.6
	Assistance	32	1.2	26	2.4
Subjective health status	Good	2381	87.1	709	66.1
	Poor	352	12.9	364	33.9
Unmet medical care	Y	759	27.8	272	25.3
	N	1974	72.2	801	74.7
Chronic disease	Y	728	26.6	789	73.5
	N	2005	73.4	284	26.5
Moderate physical activity	Y	942	34.5	351	32.7
	N	1791	65.5	722	67.3
Intense physical activity	Y	597	21.8	151	14.1
	N	2136	78.2	922	85.9
Sitting time	<8 h	1949	71.3	829	77.3
	≥8 h	784	28.7	244	22.7
Smoking	Y	1333	48.8	309	28.8
	N	1400	51.2	764	71.2
Drinking	Y	2441	89.3	717	66.8
	N	292	10.7	356	33.2

### 3.2. Comparative analysis of health-related quality of life by age

We compared and analyzed the differences in the quality of life of patients with BPH in younger adults and older adults. The health-related quality of life of the subjects (t = 14.306 ***) showed statistically significant differences. The average health-related quality of life in young & middle-aged adults was 0.79, while for older adults it was 0.55. This shows that the health-related quality of life of young & middle-aged adults was higher (Table 2).

**Table 2:** Health-related quality of life by general and medical related characteristics of participants (n = 3806).

Characteristic	Categories	Younger adults (under 65 years)				Older adults (65 years or older)		
		M(SD)	*t*	*P*	M(SD)		*t*	*P*
HRQOL		0.79 (0.406)			0.55 (0.498)		14.306***	0.000†
Education	<High school	0.63 (0.485)	–5.059***	0.000	0.50 (0.500)		–3.981***	0.000
	≥High school	0.81 (0.396)			0.62 (0.487)			
Income	< $ 2400	0.77 (0.421)	–3.065**	0.002	0.51 (0.500)		–4.760***	0.000
	≥ $ 2400	0.82 (0.387)			0.67 (0.471)			
Type of medical security	National health insurance	0.82 (0.387)	–4.013***	0.000	0.56 (0.497)		–4.539***	0.000
	Assistance	0.77 (0.421)			0.19 (0.402)			
Subjective health status	Good	0.84 (0.362)	14.804***	0.000	0.72 (0.450)		18.454***	0.000
	Poor	0.44 (0.497)			0.21 (0.411)			
Unmet medical care	Y	0.66 (0.475)	9.779***	0.000	0.38 (0.487)		6.470***	0.000
	N	0.84 (0.363)			0.60 (0.489)			
Chronic disease	Y	0.67 (0.470)	8.608***	0.000	0.49 (0.500)		7.042***	0.000
	N	0.84 (0.370)			0.71 (0.452)			
Moderate physical activity	Y	0.78 (0.412)	0.816	0.415	0.60 (0.491)		–2.326**	0.020
	N	0.80 (0.403)			0.51 (0.500)			
Intense physical activity	Y	0.76 (0.430)	2.397**	0.017	0.66 (0.477)		–2.973**	0.003
	N	0.80 (0.398)			0.53 (0.499)			
Sitting time	<8 h	0.79 (0.410)	0.423	0.672	0.55 (0.497)		0.689	0.491
	≥8 h	0.79 (0.404)			0.53 (0.500)			
Smoking	Y	0.79 (0.410)	0.656	0.512	0.56 (0.497)		–0.632	0.528
	N	0.80 (0.402)			0.54 (0.499)			
Drinking	Y	0.74 (0.441)	2.310**	0.021	0.44 (0.497)		4.990***	0.000
	N	0.80 (0.401)			0.60 (0.490)			

HRQOL = health-related quality of life.

*P < .1,

** *P* < .05,

*** *P* < .001.

† Result of independent *t*-test between young and middle-aged and older adults.

For young & middle-aged adult patients with BPH, the higher their educational attainment and income level, the higher their quality of life. Similarly, the quality of life of those who had medical security in the form of health insurance was shown to be higher. In addition, patients who thought that their subjective health state was good also had a higher quality of life. In comparison, patients who experienced unmet medical care and who had chronic diseases had a lower quality of life. The quality of life of the patients who did not participate in intense physical activities was higher; similar to the patients who do not drink alcohol who also had a higher quality of life.

In the patients with BPH in the older adult group, like those in young & middle-aged adult group, the higher the educational and income level of the patients were, the higher their corresponding quality of life. In addition, the patients whose type of medical security was health insurance had a high quality of life; the patients who felt that their subjective health state was good also had a higher quality of life score. In addition, the patients who experienced unmet medical care and who chronic diseased also had a lower quality of life. In terms of physical activity, the patients who were involved in moderate physical activities showed a higher quality of life, but those involved in intense physical activities showed a lower quality of life. Furthermore, the quality of life was higher for patients who do not drink alcohol.

### 3.3. Analysis of the factors affecting the health-related quality of life of patients with BPH by age

Factors affecting the health-related quality of life of patients with BPH according to their age were analyzed. As a result, the factors influencing the quality of life of young & middle-aged patients with BPH appeared to be educational attainment, subjective health status, unmet medical care, having a chronic disease, engagement in intense physical activity, and time spent sitting; factors affecting the quality of life of older adult patients with BPH were income level, type of medical security, subjective health status, unmet medical care, chronic disease, and alcohol consumption (Tables 3 and 4).

**Table 3:** Factors affecting health-related quality of life by age (under 65 years).

Variable (reference)		B	S.E.	Wald	p	Exp(B)	95% CI for Exp(B)	
							Lower	Upper
Education (under high school)	Over high school	0.321	0.184	3.047	0.081	1.379	0.961	1.977
Income (under $ 2400)	Over $ 2400	0.152	0.109	1.940	0.164	1.164	0.940	1.441
Type of medical security (assistance)	National health insurance	0.637	0.410	2.413	0.120	1.892	0.846	4.228
Subjective health status (good)	Poor	–1.873	0.133	198.693	0.000	0.154	0.118	0.199
Unmet medical care (no)	Yes	–1.170	0.109	114.346	0.000	0.310	0.250	0.384
Chronic disease (no)	Yes	–0.602	0.114	27.910	0.000	0.548	0.438	0.685
Moderate physical activity (no)	Yes	0.053	0.126	0.176	0.675	1.054	0.823	1.351
Intense physical activity (no)	Yes	–0.235	0.141	2.778	0.096	0.791	0.600	1.042
Sitting time (under 8 hours)	Over 8 h	–0.268	0.118	5.140	0.023	0.765	0.607	0.964
Smoking (no)	Yes	–0.043	0.106	0.166	0.684	0.958	0.778	1.179
Drinking (no)	Yes	0.120	0.166	0.527	0.468	1.128	0.815	1.561

**Table 4:** Factors affecting health-related quality of life by age (65 years or older).

Variable (reference)		B	S.E.	Wald	*P*	Exp (B)	95% CI for Exp (B)	
							Lower	Upper
Education (under high school)	Over high school	0.046	0.154	0.090	0.764	1.047	0.775	1.415
Income (under $ 2400)	Over $ 2400	0.470	0.179	6.922	0.009	1.601	1.127	2.273
Type of medical security (assistance)	National health insurance	1.072	0.562	3.636	0.057	2.921	0.971	8.792
Subjective health status (good)	Poor	–2.089	0.163	163.703	0.000	0.124	0.090	0.170
Unmet medical care (no)	Yes	–1.058	0.2172	37.752	0.000	0.347	0.248	0.484
Chronic disease (no)	Yes	–0.767	0.172	19.906	0.000	0.464	0.331	0.650
Moderate physical activity (no)	Yes	0.039	0.171	0.052	0.819	1.040	0.743	1.455
Intense physical activity (no)	Yes	0.194	0.234	0.683	0.409	1.214	0.767	1.922
Sitting time (under 8 hours)	Over 8 hours	–0.199	0.178	1.258	0.262	0.819	0.578	1.161
Smoking (no)	Yes	0.174	0.163	1.142	0.285	1.191	0.865	1.639
Drinking (no)	Yes	–0.283	0.157	3.227	0.072	0.754	0.554	1.026

In terms of the group of patients with BPH that were young and middle-aged adults, the higher their educational attainment was, the more likely they were to belong to the nondeteriorated group in the quality of life. In addition, the worse their subjective health status was, the more the unmet medical care they experienced, the more the patients were suffering from a chronic disease, the more the patients participated in intense physical activities, and the more time the patients spent sitting; the more likely they were to belong to the deteriorated group in the quality of life.

Meanwhile, in the case of older adult patients with BPH, those who had higher income and who were health insurance subscribers were more likely to belong to the nondeteriorated group in terms of quality of life. The worse their subjective health status, the more their unmet medical care, the more the patients had chronic disease, and the more the patients drank alcohol, the more likely they were to belong to the deteriorated group in terms of quality of life.

## 4. Discussion

The results of the study showed that the factors affecting the health-related quality of life of younger adult patients with BPH included educational attainment, subjective health status, unmet medical care, chronic disease, engagement in intense physical activity, and sitting time. While the factors affecting older adult patients were income level, medical security type, subjective health status, unmet medical care, chronic disease, and alcohol consumption—this shows that the factors influencing health-related quality of life differed according to age.

In the older adult patients with BPH, educational attainment, engagement in intense physical activity, and sitting time influenced quality of life. Sociodemographic characteristics were closely related to the prevalence of BPH. It is known that patients with a higher educational attainment tend to better understand the treatment process and positively participate in it. This leads to an improvement in the treatment results and, simultaneously, an improvement in the quality of life.^[23,24]^ The results of precedent studies seem to be in line with the findings of this study: the higher the educational attainment of patients, the higher their quality of life. Meanwhile, participation in intense physical activities negatively affected the quality of life of the younger adult patients with BPH, which was different from the existing study that showed that as the frequency, intensity, or amount of physical activity increased, the quality of life also increased.^[25]^ BPH constrains the daily life of patients and causes psychological pains, such as stress, thereby reducing the quality of life. However, the patients with BPH who perform a lot of physical activities have the lowest frequency of lower urinary tract symptoms; those who are usually sitting for a long period of time are at a higher risk of lower urinary tract symptoms.^[6]^ The results of this study show that engaging in moderate physical activities, rather than intense ones, have a positive effect on the quality of life of patients with BPH. Simple physical activities, such as walking, seem to be required. BPH patients often have low physical activity due to long sitting time, and it is considered necessary for them to increase their physical activity and change their lifestyle by increased walking and gymnastics. In addition, the longer the sitting time was, the lower the quality of life of the younger adult patients with BPH became. This seems to be consistent with precedent studies that show that the risk of BPH can be decreased by reducing sitting time.^[18,26]^ Therefore, it is necessary to improve the quality of life of patients with BPH through constant physical activities rather than sedentary life.

Unlike younger adults, in the older adult patients with BPH, income level, medical security type, and alcohol consumption affected the health-related quality of life. Generally, income inequality is known to affect individual health care.^[22,27,28]^ The results of this study showed that for the patients whose income level was high and who had health insurance, their quality of life was higher. This seems to coincide with the research results that for the older adult patients with BPH, those who have higher income constantly receive medication, and that the higher the patient’s share in medical expenses is, the lower their quality of life. In addition, the older adult patients with BPH who drank alcohol showed a lower quality of life. This was similar to the research results that for patients who have healthy lifestyles, such as nonengagement in smoking and alcohol consumption, have higher curative effects, and that the patients with BPH who have been continuously treated have a higher quality of life than those who are not continuously treated.^[20,29–32]^ Considering these results, it is surmised that to improve medical access across income levels for the treatment of patients with BPH, the nation should reinforce the coverage of health insurance for the treatment costs of BPH. Strengthening the coverage will enable continuous health care for older adult patients with BPH, which leads to their improved quality of life.

It was found that the common factors affecting the quality of life of patients with BPH included subjective health status, unmet medical care, and chronic disease. The patients with BPH may experience difficulties in their daily lives due to lower urinary tract symptoms, which can cause depression, anxiety, and stress, and negatively affect subjective health status.^[33–35]^ The existing studies’ findings were consistent with the results of this study that the lower the subjective health status of the patients with BPH in both the younger adult and older adult groups was, the more their quality of life decreased. BPH is often perceived as having symptoms with a low level of seriousness, unlike chronic diseases such as hypertension and diabetes. Sometimes, the patients may not feel it necessary to seek treatment because they simply think that it is a part of the inconveniences of daily life or that it will improve naturally.^[36]^ It seems that if the treatment of BPH is delayed for these reasons, the patient’s subjective health status becomes lower and their quality of life decreases. In addition, as the delay in treatment occurs, the patients with BPH cannot receive treatments at the right time or they may receive inadequate treatment, leading to a decrease in their quality of life.^[1,37]^ This seems to be in line with the results of this study that the more the patients experience unmet medical care, the more their quality of life decreases. Since the patients who have a lower educational attainment and who belong to the low-income bracket are more likely to experience unmet medical care, it is necessary to reduce the burden on the use of medical services by improving the medical accessibility of patients with BPH. Especially in older adults, as there are many cases that they consider to be a regression in physical function due to aging, it is necessary to create a medical environment where tests and treatments for the symptoms of BPH can be actively provided.

Chronic diseases, such as hypertension, diabetes mellitus, and cardiovascular disorders, are known to cause lowered reproductive function and lower urinary tract symptoms in males. In other words, chronic diseases cause BPH, and bring about troubles in daily life while reducing their quality of life.^[38,39]^ The findings of existing research are consistent with the results that chronic diseases may reduce the quality of life of patients with BPH. In addition, metabolic syndromes such as dyslipidemia and hypertension have been shown to cause lower urinary tract symptoms and BPH. Since BPH is closely related to chronic diseases, and they act as risk factors for each other, there is necessity to develop prevention measures. Particularly, obesity and hypercholesterolemia are factors that affect the morbidity of chronic diseases; it seems that weight management through exercises may help patients with chronic disease or with BPH and improve their quality of life.^[39,40]^

There are some limitations to this study. First, the quality of life of patients with BPH differed according to their socioeconomic levels. In addition, given the existing study results that those engaged in professions have a lower prevalence of BPH, future studies should conduct an analysis of the quality of life according to the occupation of the patients with BPH.^[38,41]^ Second, since an analysis of the quality of life has not been performed in the patients with BPH according to treatment method, it seems that future research may suggest therapies that can improve the quality of life of patients, through further analyses. Third, additional studies including a control group without BPH or comorbidities are needed. In this study, only patients with benign prostatic hyperplasia were analyzed, and a comparative analysis of quality of life was performed between patients with benign prostatic disease who simply had a chronic disease. In future research, the factors affecting the quality of life of BPH patients can be identified in detail by analyzing the changes in the quality of life of those with and without disease and the types of comorbidities. However, the result of this study is considered to be meaningful in that it is intended to analyze the factors influencing the quality of life of patients with BPH by age and to present a method to effectively manage the disease by age.

## 5. Conclusion

Younger adult patients with BPH are more likely to engage in economic activity than older adults, and because they have income, their burden of medical expenses is relatively low. However, their quality of life is reduced due to long sitting time and little exercise due to economic activity. Also, it seems that the quality of life of BPH patients becomes degraded if the treatment period is delayed due to simple inconvenience. Meanwhile, changes in lifestyle habits among younger adults are expected to carry over as they become older adults, helping prevent BPH. On the other hand, older adult patients with BPH often do not engage in economic activities due to retirement, so it seems that they feel burdened with the medical expenses required to manage BPH. In addition, the elderly tends to be limited in long-term management and treatment as they are prescribed drugs related to chronic diseases rather than receiving specialized treatment for BPH. In other words, it is judged that medical expenses due to the limitations of professional treatment will lower the quality of life of elderly patients with BPH.

In conclusion, in order to improve the quality of life of patients with BPH in younger adults, it is necessary to reduce the risk of chronic disease via continuous physical activities, and it seems that through this, subjective health status should change positively. Furthermore, the importance of treatment should be emphasized to encourage early treatment. On the other hand, drug treatment for patients with BPH imposes a burden on elderly patients who have lower economic capacity in the long-term. In the case of elderly patients, long-term treatment can increase their burden of medical expenses and cause their quality of life to deteriorate because income level and type of insurance affect the quality of life. Therefore, if surgical treatment is available for the treatment of older adult patients with BPH, it may be able to reduce the patient’s medical expenses. However, much time and resources must be invested in urodynamic examinations to check the progress of BPH before surgery. In addition, if surgery is delayed or unnecessary surgery is performed, the entire treatment period is increased, and the medical cost is wasted. In order to reduce patients’ burden of medical expenses, AI medical devices have recently been used to treat BPH. In particular, it is thought that the usage of AI medical devices to determine whether to perform an operation on BPH will have a positive effect on prevention of BPH and on the patient’s quality of life after treatment.

## Author contributions

Conceptualization, M.L. and K-S.L.; methodology, S.P.; software, S.P.; validation, M.L. and M.C.; resources, S.P; data curation, M.L.; writing-original draft preparation, S.P.; writing-review and editing, M.L.; visualization, M.L. and S.P.; supervision, K-S.L. and M.C.; project administration, M.L. and M.C.; funding acquisition, K-S.L. All authors read and approved the final manuscript.

Choi and Lee contributed equally to this work.

## Corrections

The funding number has been updated from (NRF-2019S1A5A2A03040304) to (NRF-2021R1I1A4A01057428). The how to cite has been updated to properly show the author order. The author contribution has been changed from “Conceptualization, M.L. and K-S.L.; methodology, S.P.; software, S.P.; validation, M.L. and M.C.; resources, K-S.L; data curation, M.L.; writing–original draft preparation, S.P.; writing–review and editing, M.L.; visualization, M.L. and S.P.; supervision, K-S.L. and M.C.; project administration, M.L. and M.C.; funding acquisition, K-S.L. All authors read and approved the final manuscript.

Lee and Park contributed equally to this work.
